# Acetate Downregulates the Activation of NLRP3 Inflammasomes and Attenuates Lung Injury in Neonatal Mice With Bronchopulmonary Dysplasia

**DOI:** 10.3389/fped.2020.595157

**Published:** 2021-02-04

**Authors:** Qian Zhang, Xiao Ran, Yu He, Qing Ai, Yuan Shi

**Affiliations:** ^1^Department of Neonatology, Children's Hospital of Chongqing Medical University, Chongqing, China; ^2^Chongqing Key Laboratory of Pediatrics, Chongqing, China; ^3^Ministry of Education Key Laboratory of Child Development and Disorders, National Clinical Research Center for Child Health and Disorders, China International Science and Technology Cooperation Base of Child Development and Critical Disorders, Chongqing, China

**Keywords:** GPR43, microbial communities, inflammasome, acetate, bronchopulmonary dysplasia

## Abstract

**Background:** Bronchopulmonary dysplasia (BPD) is a common pulmonary complication in preterm infants. Acetate is a metabolite produced by the gut microbiota, and its anti-inflammatory function is well known. The role of acetate in BPD has not been studied. Here, we investigate the effects of acetate on lung inflammation and damage in mice model of BPD.

**Objective:** To investigate the role of acetate in the development of BPD.

**Methods:** C57BL/6 mice were randomly divided into three groups on the 3rd day after birth: room air group, hyperoxia group, and hyperoxia + acetate (250 mM, 0.02 ml/g) group. The expression of inflammatory factors was determined by ELISA and RT-PCR, and NLRP3 and caspase-1 were detected by Western blot. High-throughput sequencing was used to detect bacterial communities in the mice intestines.

**Results:** After acetate treatment, the expression levels of TNF-α, IL-1β, IL-18, NLRP3, and caspase-1 were significantly reduced, while the expression of GPR43 was increased. In the BPD mice treated with acetate, the proportion of Escherichia-Shigella was lower than in placebo-treated BPD mice, while the abundance of Ruminococcus was increased.

**Conclusions:** These results indicate that acetate may regulate intestinal flora and reduce inflammatory reactions and lung injury in BPD. Therefore, acetate may be an effective drug to protect against neonatal BPD.

## Introduction

Bronchopulmonary dysplasia (BPD) is the most common pulmonary complication of very premature birth (1), and its prevalence is increasing, most likely due to the prolonged survival of preterm infants (2). BPD is characterized by stunted development involving impaired alveolar and vascular growth (3, 4). In addition to preterm birth, inflammation and infection are all related to the pathogenesis of BPD (4, 5). Pro-inflammatory factors such as tumor necrosis factor (TNF)-α and interleukin (IL)-1β have been associated with the development of BPD (3, 6, 7). Inflammasomes are a series of multiprotein complexes that modulate the innate immune responses (8). NLRP3 is the most commonly studied protein in the inflammasomes and activated by infection, tissue lesion and oxidative stress. Meanwhile, activation of NLRP3 could promote IL-1β and IL-18 secretion (9, 10). Many studies have shown that inflammasome activation is associated with various inflammatory disorders. Previous studies have shown that NLRP3 inflammasome plays an important role in the pathogenesis of BPD (11). Therefore, the regulation of NLRP3 activation may be a therapeutic target of BPD.

Acetate is produced by the intestinal flora, and well known for its anti-inflammatory function (12, 13). In most cases, acetate acts by binding to its receptor. G protein-coupled receptor 43 (GPR43) is the natural receptor for acetate (14). Antunes et al. (12) demonstrated that acetate activated and regulated type 1 responses in lung epithelial cells by GPR43 to prevent RSV infection. Another study showed that acetate binds to GPR43 to reduce the inflammasome in lipopolysaccharide (LPS)-induced endotoxemia through sAC-PKA (15). Despite the acetate have shown anti-inflammatory effects by regulating NLRP3 in several peritonitis models, its role in BPD remains unknown.

In this study, we investigated the effects of acetate on inflammation and lung damage in mice model of BPD and found that acetate intervention could alter the abundance of gut microbiota.

## Materials and Methods

### Animal Model and Experimental Protocol

This study was approved by the Ethics Committee for Animal Protection and Use of Chongqing Children's Hospital. All C57BL/6 mice were purchased from Chongqing Medical University. Mice were housed under environmentally controlled conditions, and pregnant mice were kept in separate cages. All studies used newborn mice, and the number of pups in each litter was adjusted to 5–7 mice in each group to minimize the impact of nutritional differences on lung development. All mice were weighed on the day of birth and during modeling. According to the experimental conditions, newborn mice on postnatal day 3 (P3) were randomly divided into 3 groups (*n* = 5–7/group): room air (21% O_2_) + placebo (phosphate-buffered saline, PBS) group, hyperoxia (85% O_2_) + placebo (PBS) group, and hyperoxia (85% O_2_) + acetate group. During continuous exposure to hyperoxia, acetate (250 mM, 0.02 ml/g) (15) was given daily for 14 consecutive days. The continuous exposure to 85%O_2_ was achieved in a sealed oxygen chamber, and the oxygen concentration inside was continuously monitored with an oxygen meter. To avoid oxygen poisoning of nursing mothers, these mice were exchanged between the hyperoxia and room air conditions every 24 h.

### Mouse Lung Tissue Harvesting

The mice were anesthetized by intraperitoneal injection of pentobarbital sodium on P17. The left lung was completely fixed in 4% paraformaldehyde and then embedded in paraffin. The right lung was immediately stored in −80°C for the measurement of gene expression, proteins, and cytokine.

### Lung Histopathology

Standard inflation was used in generating the lung histopathology as previously described (6). 4%Paraformaldehyde-fixed lung tissue was embedded in paraffin and cut into 4 μm sections. Lung tissues sections were stained with hematoxylin and eosin (H&E) for morphological analysis by optical microscopy at a magnification of 200x (Nikon, Japan). Six fields of view were randomly selected on each tissue section. Alveolar development was estimated using the mean linear intercept (MLI) and radial alveolar count (RAC) (11, 16). The MLI is used as a method to estimate volume-to-surface ratio of acinar airspaces complex. The RAC is used to quantify alveolarization and estimate lung maturation. All images were evaluated by two blinded investigators. Data analyses were performed using Image-ProPlus software.

### RNA Isolation and Quantitative Real-Time PCR

Total RNA was separated and purified using the TRIZOL reagent (Invitrogen). The A260/A280 ratio was measured to determine the RNA concentration. Takara Primescript RT (China) kits were used for reverse transcription according to the instructions.

The primers were as follows: IL-1β (Forward: TGGTGTGTGACGTTCCCATT Reverse: CAGCACGAGGCTTTTTTGTTG), TNF-α (Forward: GACGTGGAACTGGCAGAAGAG Reverse: CAGGAATGAGAAGAGGCTGAGAC), NLRP3 (Forward: ATTACCCGCCCGAGAAAGG Reverse: CATGAGTGTGGCTAGATCCAAG), GPR43 (Forward: CTTGATCCTCACGGCCTACAT Reverse: CCAGGGTCAGATTAAGCAGGAG), GAPDH (Forward: AGGTCGGTGTGAACGGATTTG Reverse: GGGGTCGTTGATGGCAACA). Reactions were performed in the Bio-Rad CFX Real-Time PCR system. The software of this instrument was used to compute the threshold cycle (Ct) values of all genes. The expression of each gene was normalized to that of GAPDH.

### Cytokine Measurements

The inflammatory cytokine levels in lung homogenates of mice were evaluated. According to the manufacturer's instructions, the pulmonary concentrations of IL-18 (Xinbosheng China), IL-1β (Sizhengbai China), and TNF-α (Sizhengbai China) were measured by mouse cytokine enzyme-linked immunosorbent assay (ELISA) kits.

### Western Blotting Analysis

We extracted proteins from whole lung tissues. Western blot was performed according to a protocol described by Chen et al. (6). Antibodies recognizing NLRP3 (Abcam, UK) and caspase-1-p20 (GeneTex, USA) were used.

### Microbiome Analysis

After the mice were anesthetized by intraperitoneal injection of pentobarbital sodium on P17, feces were collected under sterile conditions and stored in −80°C. According to the manufacturer's instructions, QIAamp FAST DNA Stool Mini Kit (Qiagen, Hilden, Germany) was used to extract bacterial DNA. PCR was used to amplify the 16S ribosomal DNA variable V3-V4 region, and the primer sequences were 338F (5′-ACTCCTACGGGAGGCAGCAG-3′) and 806R (5′-GGACTACHVGGGTWTCTAAT-3′). The original data obtained by Miseq sequencing were processed and optimized. Operational taxonomic units (OTUs) were clustered at 97% similarity. At the phylum and genus levels, we analyzed the relative abundance of each OTU. Data analyses were run on the free online platform of Majorbio Cloud Platform (www.majorbio.com).

### Statistical Analysis

SPSS version 22.0 (SPSS Inc., USA) was used for statistical analysis. Data between multiple groups were analyzed by one-way analysis of variance (ANOVA). *P* value < 0.05 was considered statistically significant.

## Results

### Body Weight of Neonatal Mice

**Figure 1:**
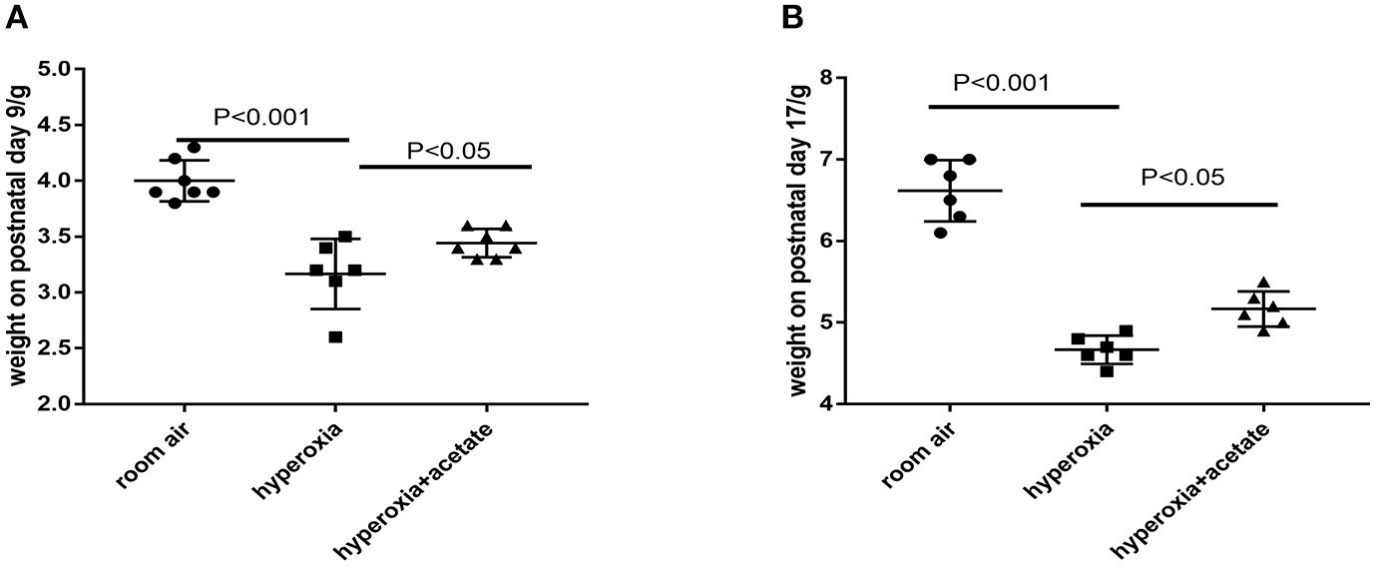
Body weight of neonatal mice. **(A)** Acetate-treated mice in the hyperoxia group had a significantly higher body weight on postnatal day 9 than non-acetate treated mice (*P* < 0.001). **(B)** The body weight on postnatal day 17 (*P* < 0.001). *n* = 8 mice/group. Data are expressed relative to the control group at each time point as the mean ± SEM.

Compared with the room air group, the body weight of hyperoxia + placebo group was obviously decreased at P9. However, the body weight of hyperoxia + acetate group increased at P9 compared to that of the hyperoxia + placebo group (Figure 1A). Similar weight differences were observed at P17 (Figure 1B), but the difference in weight could not be completely reversed.

### Acetate Attenuates Morphological Changes of the Lung

**Figure 2:**
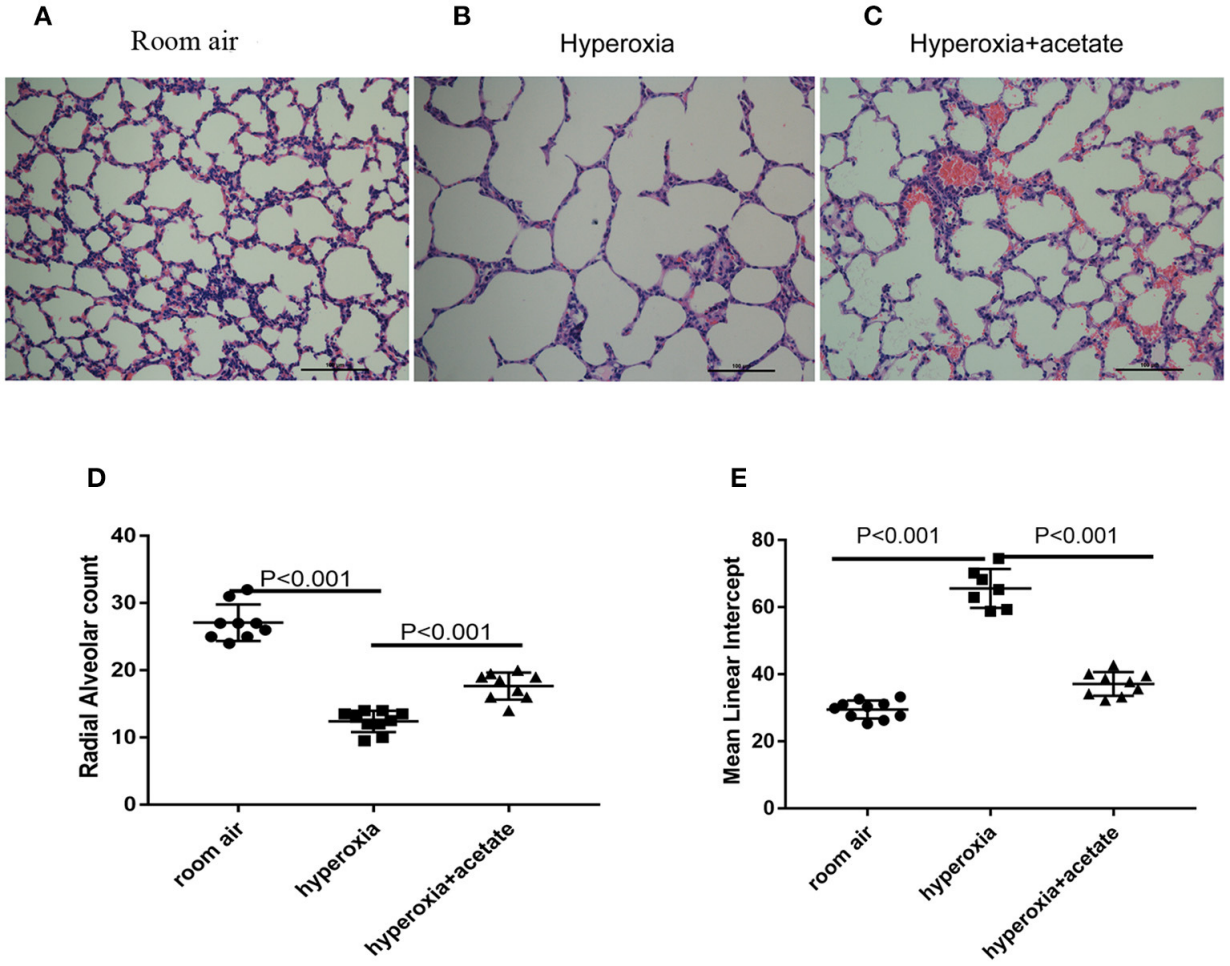
Acetate attenuates morphological changes. Hyperoxia exposure led to marked alveolar simplification as shown by H&E staining and by RAC and MLI assessment. Acetate treatment improved the hyperoxia-induced impairment of alveolar growth. **(A–C)** Representative H&E staining (light microscopy, ×200) of lung tissue slides from each group. Scale bars = 100 μm. **(D)** Semiquantitative pathological determination of RAC in lung tissues. **(E)** Semiquantitative pathological determination of MLI in lung tissues. The values represent the mean ± SD; *n* = 6 mice/group.

H&E staining was used to examine lung morphology (Figure 2). In the room air group, the lung exhibited complete lung structures with a normal alveolar epithelium and alveolar septum (Figure 2A). However, after 14 days of hyperoxia exposure, the alveoli of the mice were enlarged (Figure 2B), the RAC of per unit area significantly reduced, while the MLI significantly increased (Figures 2D,E). These data showed that exposure to hyperoxia increased alveolar damage and led to alveolar simplification. Alveolar simplification and alveolar septal improvement were observed in the hyperoxia + acetate group compared to the hyperoxia group (Figure 2C). These results demonstrated that acetate therapy could resist high O_2_-induced lung injury and partly restore high O_2_-induced alveolar simplification.

### Acetate Decreases Inflammatory Factors in the Mouse Lung

**Figure 3:**
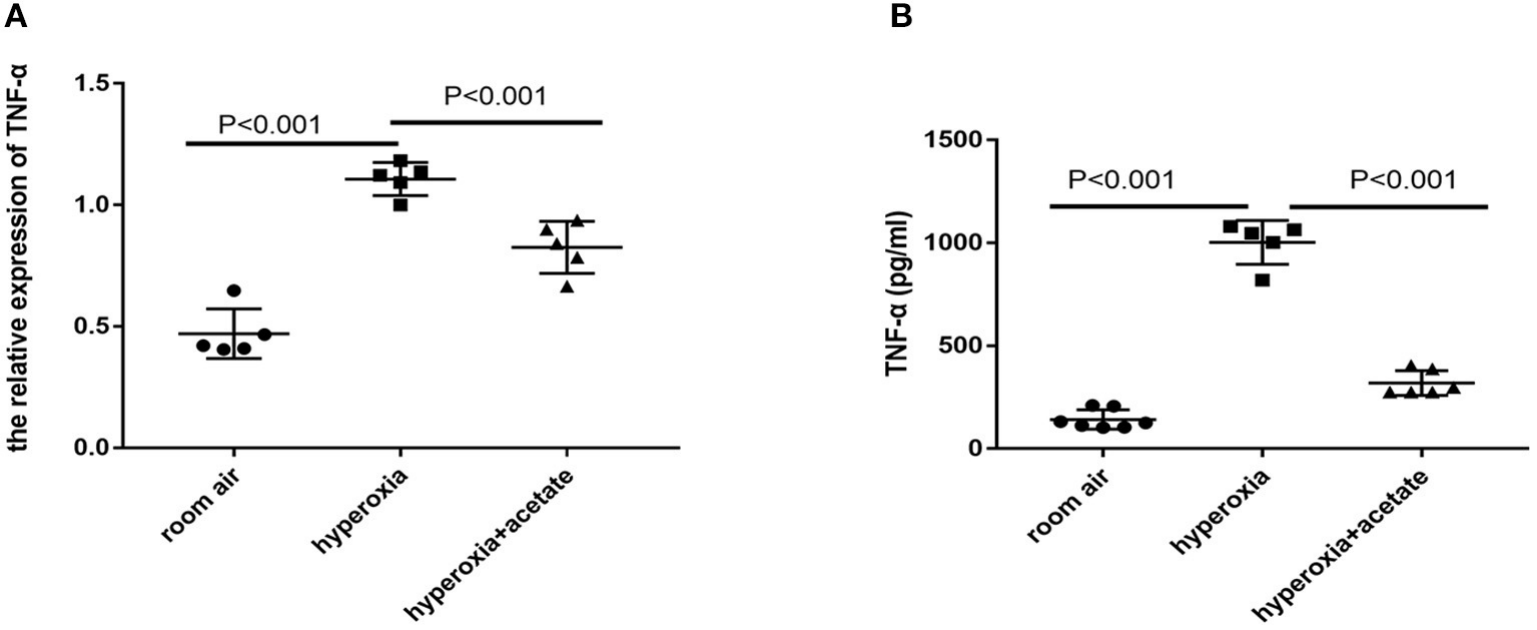
Acetate decreases pro-inflammatory factors in the mouse lung. The pulmonary mRNA levels of TNF-α **(A)**. The pulmonary protein levels of TNF-α **(B)**. *n* = 5–7/group from 3 independent experiments; data are presented as the means ± SD.

RT-PCR and ELISA were used to detect the expressions of inflammatory cytokines in lung tissues. Compared with the room air group, the expression of TNF-α was significantly increased after 14 days of exposure to the high O_2_ level. However, in the hyperoxia + acetate group, the expression of TNF-α was significantly lower than the hyperoxia group (Figures 3A,B). These results indicated that acetate treatment decreases the expressions of TNF-α in the lung caused by hyperoxia.

### Acetate Reduces the Expression of NLRP3 Inflammasome-Related Proteins and GPR43

**Figure 4:**
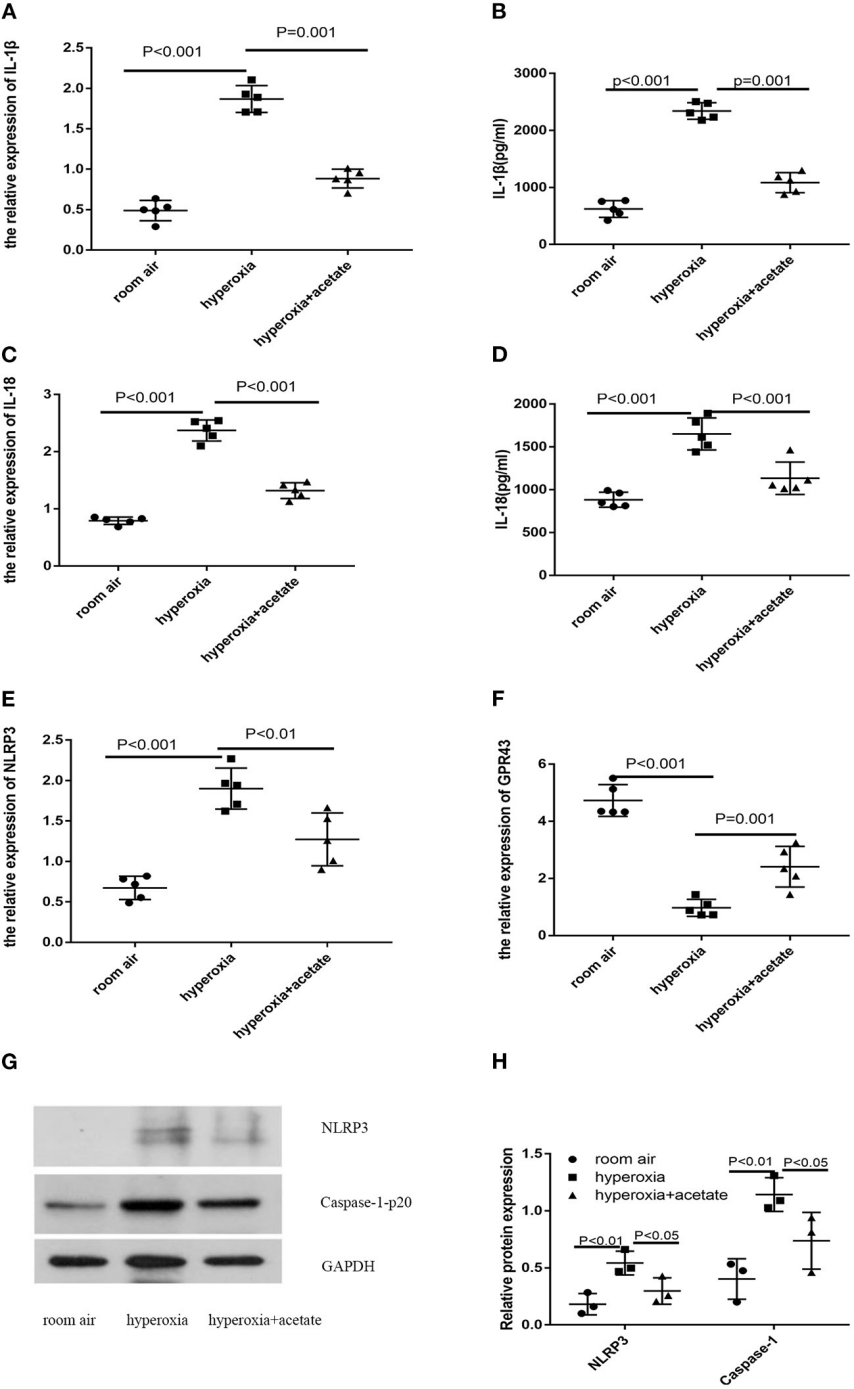
Acetate decreases NLRP3 inflammasome-related protein and GPR43 expression. The pulmonary mRNA levels of IL-1β **(A)**, IL-18 **(C)**, NLRP3 **(E)** and GPR43 **(F)**. The pulmonary protein levels of IL-1β **(B)**, IL-18 **(D)**, NLRP3 **(G,H)** and caspase-1 **(G,H)**. *n* ≥ 3/group from 3 independent experiments; data are presented as the means ± SD.

To assess the effect of acetate on the lung NLRP3 inflammasome-related cytokines IL-1β and IL-18, lung tissues were examined by RT-PCR and ELISA. Compared with the room air group, the expression of IL-18 in hyperoxia group was significantly increased (Figures 4C,D). However, the expression of IL-18 in the hyperoxia + acetate group was significantly lower than the hyperoxia group. Similar results were also observed for IL-1β (Figures 4A,B). Then, RT-PCR and Western blot were used to detect the expressions of NLRP3 inflammasome and caspase-1 in lung tissue.

Compared with the room air group, the expressions of NLRP3 inflammasome and caspase-1 were significantly increased in the hyperoxia group, and these effects could be partially reversed by the acetate treatment (Figures 4E,G,H).

Next, we investigated whether acetate could alter the expression level of GPR43 in newborn mice exposed to high oxygen. Compared with the control group, high oxygen exposure reduced the expression of GPR43 in lung tissue (Figure 4F). Acetate treatment could partially increase the expression of GPR43. These data showed that acetate affects the expression of NLRP3 and GPR43 in lung tissue after hyperoxia exposure.

### Acetate Treatment Affects the Composition of the Gut Microbiota in Mice Exposed to Hyperoxia

**Figure 5:**
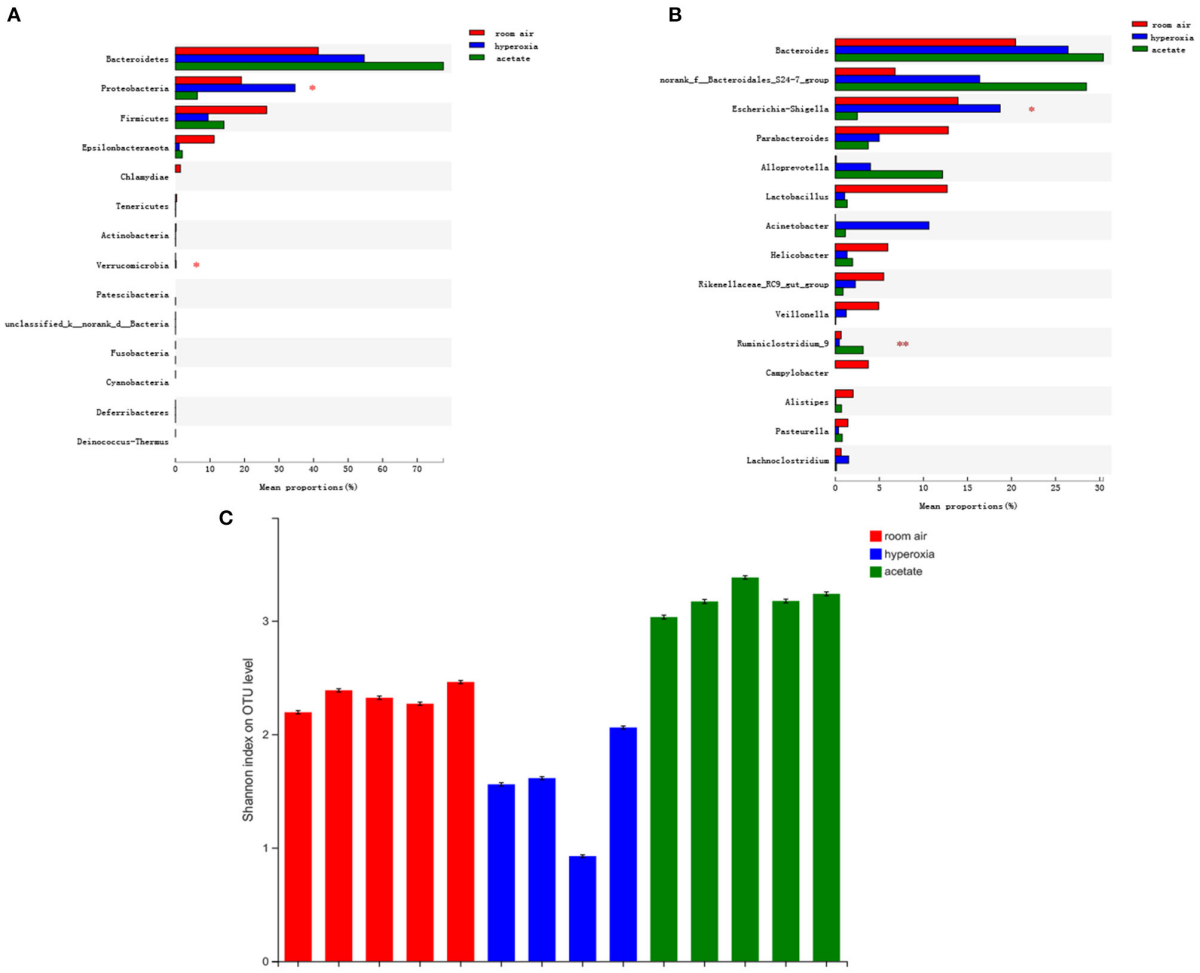
Acetate treatment affects the composition of the gut microbiota in mice exposed to hyperoxia. The relative abundance of microbial communities in the gut of the 3 groups at the phylum **(A)** and genus **(B)** levels. For **(A,B)**, relative abundance >1%, *P < 0.05 and **P < 0.01. Assessment of the diversity of microbial communities in the intestinal contents **(C)** of the 3 groups by the Shannon Diversity Index (room air: red, hyperoxia: blue, acetate: green).

To investigate whether acetate could change the composition of intestinal flora, we used the 16S rRNA method to analyze the intestinal contents to determine the changes in intestinal flora. The bacterial compositions of the three groups of mice were significantly different at the phylum and genus levels. Compared with the room air group, the abundance of Proteobacteria in the gut was significantly higher in the hyperoxia group (*P* < 0.05) (Figure 5A), while no significant difference was observed in the proportion of Bacteroidetes and Firmicutes in the gut. In the hyperoxia+acetate group, the abundance of Proteobacteria was decreased. In addition, at the genus level (Figure 5B), we found an increase in the relative abundance of Escherichia/Shigella in the hyperoxia group compared with the room air group (*P* < 0.001), while the relative abundance of Ruminococcus was significantly decreased (*P* < 0.05). In the hyperoxia+acetate group, the relative abundance of Escherichia/Shigella was decreased, while the relative abundance of Ruminococcus was significantly increased.

## Discussion

BPD is a chronic lung disease of prematurity and it is usually accompanied by delayed pulmonary hypertension, persistent pulmonary dysfunction, developmental delay, and neurocognitive problems in later life (17, 18). In this study, hyperoxia activated the NLRP3-IL-1β axis and induced the pathological features of BPD in newborn mice. We found that acetate downregulated the expression levels of NLRP3-related proteins, alleviated lung inflammation, and improved simplification of alveoli. Acetate intervention could also alter the abundance of gut microbiota.

Lung injury induced by hyperoxia exposure is the most common model of BPD in neonatal mice. Impaired alveolarization and angiogenesis disorders are the main pathological features of BPD (1, 19). Studies have confirmed that lung development of mice undergoes five stages: embryonic, pseudoglandular, canalicular, saccular, and alveolar stages, suggesting that the process of alveolarization has a similar time sequence as that in humans (20). However, unlike the development of the human embryonic lung, neonatal mice lung tissue only completes gross morphogenesis, and alveolarization begins 3 days after birth (21, 22). In the present study, a mouse model of BPD was successfully established by selecting 3-day-old newborn mice and continuously exposing them to 85% O_2_ for 14 days. H&E staining of the lung tissue demonstrated simplification of alveoli after high O_2_ exposure, which is consistent with the pathological features of BPD. NLRP3 activation results in excessive inflammation, which is associated with a variety of inflammatory disorders, including type 1 diabetes, hyperinflammation following influenza infection, asthma and gout (23–26). NLRP3, ASC and pro-caspase-1 are the main components of the NLRP3 inflammasome (10). Infection, tissue lesions and oxidative stress can activate the NLRP3 inflammasome. Activation of this complex processes pro-caspase-1 into an active 20 kDa fragment, which can enzymatically cleave pro-IL-1β into mature IL1β, and process pro-IL-18 into mature IL-18 (27). This process plays an important role in the initiation of inflammation (8). Liao's research showed that NLRP3 activation is one of the primary causes of BPD (11). In the present study, after 14 days of hyperoxia exposure, the levels of NLRP3, caspase-1, IL-1β, IL-18, and TNF-α were significantly increased compared to the room air group. After acetate treatment, the expression levels of NLRP3, caspase-1, IL-1β, IL-18, and TNF-α were significantly decreased. In BPD mice, the increased accumulation of macrophages can be observed (11). After RSV infection, acetate intervention reduces the recruitment of inflammatory cells including macrophages and lymphocytes into the lungs of mice, thus reducing lung inflammation (12, 28). In addition, acetate reduces the activation of NLRP3 through ubiquitination and autophagy and plays an anti-inflammatory role in peritonitis and endotoxemia models (15). Our results are consistent with these studies and suggested that acetate intervention may reduce lung inflammation. However, the effect of acetate intervention on inflammatory cells in BPD needs to be further explored *in vitro*. Subsequently, we explored the mechanism by which acetate reduces NLRP3 expression. Short-chain fatty acids (SCFAs) are metabolites derived from the gut flora, which are produced by the fermentation of indigestible dietary fiber (12) and mainly consist of acetate, propionate, and butyrate. These fatty acids are absorbed into the circulatory system and act through binding G protein-coupled receptors (GPR43 and GPR41) or inhibiting histone deacetylases (HDACs) (29, 30). It is well known that acetate can activate GPR43, and this receptor was originally recognized in colitis (29). Activation of GPR43 can reduce inflammation, and in this manner, SCFAs protect the colon from injury. Our results also indicated that after 14 days of hyperoxia, the expression of GPR43 in the newborn mice decreased and this decrease would be improved by acetate treatment. However, because GPR43 is widely expressed on the membranes of different cells, such as macrophages and epithelial cells (31), it is unclear which cell type plays a key role; further research is needed. In addition, the causal relationship between GPR43 and NLRP3 requires further investigation. Thorburn et al. demonstrated that acetate specifically activates GPR43 and subsequently decreases Ca2^+^ to activate downstream signals (32). However, an increase in Ca2^+^ can further promote the activation of NLRP3 (33). It can be concluded that after activation of GPR43 by acetate, NLRP3 activation is inhibited by decreasing Ca2^+^.

The colonization pattern of microbiota in very low birth weight (VLBW) infants is significantly different from that in healthy full-term infants (34, 35). Because acetate, a main component of SCFAs, significantly ameliorates inflammation in BPD, we speculate that the intestinal microbiota may affect the susceptibility and/or severity of BPD. Previous studies have demonstrated that gut microbiota may affect the immune response of the lungs (36–39), which indicates that gut microbiota may affect the severity of BPD by regulating systemic and lung inflammation. As previous reports, Escherichia/Shigella from the Enterobacteriaceae were significantly increased in infants with BPD (40). Escherichia-Shigella belongs to the Proteobacteria phylum and produces LPS (41). Since the LPS of Escherichia/Shigella may activate the immune system and cause inflammation (41), the increase in the relative abundance of Escherichia/Shigella in the hyperoxia group compared to the acetate group shown in our study suggests that higher levels of inflammation may have occurred in the hyperoxia group. Ruminococcaceae, which is an important source of SCFA production (42), also decreased after hyperoxia exposure but increased after administration of acetate in our study, these results are consistent with previous studies showing that Ruminococcaceae have anti-inflammatory effects (43–45) and can ameliorate lung injury (45, 46). All of these findings suggested that intestinal flora may be involved in the protective effect of acetate against lung injury.

In our study, some limitations are to be mentioned. (i) Although we found that acetate could change the composition of intestinal flora in the model of hyperoxia-induced lung injury, the combined effects of hyperoxia + acetate were considered and the independent effect of acetate on the perinatal intestinal was not considered. At present, no studies have investigated the effects of acetate treatment during the perinatal period on gut microbiota. However, a study on hypertension showed that acetate changed the gut microbiota composition, increasing the levels of bacteria from the Bacteroides genus (47). Thus, acetate-independent effects on the perinatal microbiome should be added into the future investigations. (ii) Studies have proven that male neonatal are more susceptible to hyperoxic lung injury (48). In our experimental design, sex was not taken into account as a variable, which may only reflect part of the role of acetate.

In summary, our research showed that acetate treatment can alleviate lung injury after hyperoxia exposure by inhibiting the expression of NLRP3-related proteins, which indicates that acetate treatment may be helpful for the treatment or prevention of BPD. The mechanism remains to be elucidated.

## Data Availability Statement

The raw data supporting the conclusions of this article will be made available by the authors, without undue reservation.

## Ethics Statement

The animal study was reviewed and approved by Ethics Committee for Animal Protection and Use of Chongqing Children's Hospital.

## Author Contributions

QZ, YH, and YS contributed to the concept and design of the study. QZ performed the main experiments. XR performed a portion of the statistical analysis. QZ wrote the first draft of the manuscript. YS, YH, and QA have revised this manuscript. All authors have approved the final version before submission.

## Conflict of Interest

The authors declare that the research was conducted in the absence of any commercial or financial relationships that could be construed as a potential conflict of interest.
